# EEG-fMRI Signal Coupling Is Modulated in Subjects With Mild Cognitive Impairment and Amyloid Deposition

**DOI:** 10.3389/fnagi.2021.631172

**Published:** 2021-04-23

**Authors:** Lars Michels, Florian Riese, Rafael Meyer, Andrea M. Kälin, Sandra E. Leh, Paul G. Unschuld, Roger Luechinger, Christoph Hock, Ruth O'Gorman, Spyros Kollias, Anton Gietl

**Affiliations:** ^1^Department of Neuroradiology, Clinical Neuroscience Center, University Hospital Zurich, Zurich, Switzerland; ^2^Department of Geriatric Psychiatry, Psychiatric University Hospital Zurich (PUK), Zurich, Switzerland; ^3^University Research Priority Programs (URPP) ≪Dynamics of Healthy Aging≫, University of Zurich, Zurich, Switzerland; ^4^Institute for Regenerative Medicine (IREM), University of Zurich, Zurich, Switzerland; ^5^Geriatric Psychiatry, Geneva University Hospitals (HUG), Geneva, Switzerland; ^6^Institute of Biomedical Engineering, University and Eidgenössische Technische Hochschule (ETH) Zurich, Zurich, Switzerland; ^7^Neurimmune AG, Schlieren, Switzerland; ^8^Center for Magnetic Resonance Research, University Children's Hospital Zurich, Zurich, Switzerland

**Keywords:** mild cognitive impairment, amnestic, Alzheimer's, dementia, functional MRI, electroencephalography

## Abstract

Cognitive impairment indicates disturbed brain physiology which can be due to various mechanisms including Alzheimer's pathology. Combined functional magnetic resonance imaging (fMRI) and electroencephalography (EEG) recordings (EEG-fMRI) can assess the interplay between complementary measures of brain activity and EEG changes to be localized to specific brain regions. We used a two-step approach, where we first examined changes related to a syndrome of mild cognitive impairment irrespective of pathology and then studied the specific impact of amyloid pathology. After detailed clinical and neuropsychological characterization as well as a positron emission tomography (PET) scans with the tracer 11-[C]-Pittsburgh Compound B to estimate cerebral amyloid deposition, 14 subjects with mild cognitive impairment (MCI) (mean age 75.6 SD: 8.9) according to standard criteria and 21 cognitively healthy controls (HCS) (mean age 71.8 SD: 4.2) were assessed with EEG-fMRI. Thalamo-cortical alpha-fMRI signal coupling was only observed in HCS. Additional EEG-fMRI signal coupling differences between HCS and MCI were observed in parts of the default mode network, salience network, fronto-parietal network, and thalamus. Individuals with significant cerebral amyloid deposition (amyloid-positive MCI and HCS combined compared to amyloid-negative HCS) displayed abnormal EEG-fMRI signal coupling in visual, fronto-parietal regions but also in the parahippocampus, brain stem, and cerebellum. This finding was paralleled by stronger absolute fMRI signal in the parahippocampus and weaker absolute fMRI signal in the inferior frontal gyrus in amyloid-positive subjects. We conclude that the thalamocortical coupling in the alpha band in HCS more closely reflects previous findings observed in younger adults, while in MCI there is a clearly aberrant coupling in several networks dominated by an anticorrelation in the posterior cingulate cortex. While these findings may broadly indicate physiological changes in MCI, amyloid pathology was specifically associated with abnormal fMRI signal responses and disrupted coupling between brain oscillations and fMRI signal responses, which especially involve core regions of memory: the hippocampus, para-hippocampus, and lateral prefrontal cortex.

## Introduction

Central to a functional brain physiology is the interplay between neuronal activity and metabolic demand, which is closely co-regulated in the brain. Using simultaneous EEG and fMRI enables to directly link electrophysiological scalp-recorded EEG activity to cortical and subcortical fMRI blood-oxygen-level-dependent (BOLD) signal responses. While the precise physiological relationship between the resting EEG amplitudes and BOLD signal remains vague (Laufs, 2008), the two signals co-vary in terms of their temporal fluctuation (following appropriate convolution to account for the lag and the lower frequency range of the BOLD signal). This suggests that both signals are functionally coupled. Hence, the term “coupling” as used in this study does not describe the physiological mechanism underlying their coupling, but the correlation reflecting the statistical similarity between their (convolved) time courses.

Several studies have repetitively identified a thalamocortical circuit associated with alpha band oscillations (Goldman et al., 2002; Moosmann et al., 2003; Feige et al., 2005; Goncalves et al., 2006; de Munck et al., 2007; Difrancesco et al., 2008; Tyvaert et al., 2008b). Furthermore, Scheeringa et al. found the so-called default mode network (DMN) (Raichle et al., 2001; Raichle and Snyder, 2007) to be (inversely) correlated with frontal midline theta (4–8 Hz) power (Scheeringa et al., 2008). Apparently, different EEG rhythms seem to reflect different functional networks (Laufs et al., 2003a, 2006; Mantini et al., 2007). A reliable observation of concurrent EEG-fMRI studies is that for lower frequencies (delta-alpha), EEG power is negatively correlated to the BOLD signal, signifying that these EEG signals become elevated with lowered neuronal activity in related brain regions.

Research using simultaneous EEG-fMRI during the resting state has been mostly performed in healthy children (Luchinger et al., 2012), adolescents (Luchinger et al., 2011), and adults (Debener et al., 2006; Michels et al., 2010). A central observation of the developmental studies was the lack of a thalamocortical coupling (in the alpha and beta band) in children. So far, changes with aging in the coupling of EEG rhythms to the BOLD signal in healthy elderly has only been studied in one study (Balsters et al., 2013). The authors compared two groups with different mean age (71 vs. 73 years) and identified four (out of 26) resting-state networks showing fMRI and EEG-fMRI group differences (1: anterior DMN, 2: frontal-parietal network (FPN: comprising the angular gyrus, supramarginal gyrus, superior parietal lobe, supplementary motor area, ventral premotor cortex, and some prefrontal regions), 3: middle frontal, and 4: postcentral gyri). Seven resting-state networks revealed only EEG-fMRI differences, highlighting that simultaneous EEG-fMRI is more sensitive to identify age-related neural changes compare to the fMRI. Interestingly, activity within some EEG-fMRI resting-state networks was better explained by neuropsychological measures [Mini mental status exam (MMSE) and Stroop test] than age. The simultaneous EEG-fMRI method has not been applied in people suffering from cognitive problems, such as MCI or Alzheimer's dementia (AD), except of in one study (Brueggen et al., 2017). In a final sample of 14 healthy controls (HCS) and 14 patients with mild AD, Brueggen et al. compared EEG-fMRI coupling differences using a 32-channel MRI-compatible EEG device. Focusing on the alpha band and occipital electrodes, the main finding was a reduced positive association between the fMRI signal and upper alpha band power of the visual cortex in the frontal cortex, inferior temporal gyrus, and thalamus in the patient group. In addition, negative correlations were weaker in the lower alpha band in the hippocampus as well in the cerebellum and putamen.

In this study, we aimed to compare fMRI, and EEG-fMRI signal differences between heathy elderly and individuals with MCI using high-density (64 channel) EEG recordings. We applied a regional and a validated whole-brain EEG-fMRI signal correlation approach (Jann et al., 2009; Michels et al., 2010, 2012; Luchinger et al., 2011, 2012) to characterize frequency-band coupling differences with the maximal spatial sensitivity and extent. We hypothesized abnormal coupling in the described networks (DMN and FPN) in patients with MCI, as sign of altered neurophysiological processing.

In a second approach, we aimed to dissociate fMRI and EEG-fMRI signal coupling differences between individuals with significant amyloid-beta deposition (MCI and HCS combined) from healthy controls with low amyloid deposition. We hypothesized that memory and cognitive-control related areas would show alterations in fMRI and EEG-fMRI signal coupling. Candidate regions were regions in which functional and structural alterations associated with MCI and/or high amyloid deposition have been described, such as the posterior cingulate cortex (PCC) (Hedden et al., 2009; Rane et al., 2018; Hampton et al., 2020) or the hippocampus (Sperling et al., 2009; Sheline et al., 2010a; Lim et al., 2013; Sepulcre et al., 2013; Vannini et al., 2013; Huijbers et al., 2015; Hanseeuw et al., 2016; Jacobs et al., 2018; Quevenco et al., 2019). Our analysis approach focuses on absolute (EEG) power and does not include e.g., (EEG) connectivity-derived measures—that are known to be altered in MCI and AD (Jelic et al., 1996; Rossini et al., 2006)—as the setup of this multimodal study is rather complex: four methods: fMRI and EEG-fMRI, amyloid PET, ApoE-Genotyping; two diagnostic groups; regional vs. local EEG-fMRI signal coupling differences.

## Methods

### Participants

All individuals of this sub-study participated in two longitudinal cohort studies measuring factors associated with cognitive performance in the elderly, and parts of this dataset have already been published (Riese et al., 2015; Michels et al., 2016, 2017). Cognitively healthy subjects needed to fulfill the following criteria: MMSE score ≥ 27 as well as a clinical and neuropsychological examination not suggestive of MCI or any cognitive disorders. Amnestic MCI (aMCI) was diagnosed by standard criteria (Winblad et al., 2004). Participants were ≥ 55 years of age and did not show significant depression, as assessed by a specialist in psychiatry. Exclusion criteria were medication or drug abuse that may affect cognition. In addition, subjects with MCI were excluded if they had evidence of a clinically significant neurological, psychiatric, or general medical condition that would significantly disturb cognition. Similarly, individuals were omitted if they had contraindications for MRI or PET imaging or cerebrovascular lesions in memory-related brain structures.

### Clinical and Neuropsychological Assessment

The neuropsychological and clinical examination have been published previously (Riese et al., 2015; Michels et al., 2016, 2017). All participants received a detailed clinical assessment including medical history as well as physical and neurological examination. Mini Mental Status Examination was used to assess global cognition (Folstein et al., 1975). The CERAD- test battery was applied for neuropsychological assessment (Morris et al., 1989; Thalmann et al., 1997) including digit span, trail making tests A and B, letter fluency, category fluency, Stroop interference, Boston naming test, verbal learning, recall and recognition, figure copy and recall. In addition, participants did the German version of the Rey Auditory Verbal Learning Test (VLMT) (Helmstaedter et al., 2001), the Visual Paired Associates test from the Wechsler Memory Scale Revised (WMS-R) (Haerting et al., 2000). All raw values were z-transformed according to test-specific normative data adjusted for age, gender, and education where possible. Cognitive impairment was defined if at least one score per domain was 1.5 standard deviations below group means. The mean period between the neuropsychological evaluation and MRI scanning was 8.6 months for HCS (range: 2–13 months) and 3.6 months for aMCI (range: 0–8 months).

### Analysis of Demographic and Clinical Data

Categorical data were analyzed using Chi-square (χ^2^) test [e.g., PiB-positivity (yes/no) or APOE status (carrier/non-carrier)]. *T*-Tests and Mann-Whitney *U*-tests were applied to compare normally/not normally distributed continuous variables (e.g., age and test scores).

### 11-[C]-Pittsburgh Compound B-PET

The full methods to assess cerebral amyloid-load were described previously (Steininger et al., 2014; Riese et al., 2015; Michels et al., 2017) and shall only be described in brief: Following antecubital injection of ~350 MBq of [11C]-PiB, a dynamic scan was acquired for > 70 min or a static image 50–70 min post-injection. Time activity curves were acquired from predefined volumes of interest using a maximum probability atlas (Hammers N30R83) [37, 38] that were intersected with the individuals' gray matter masks. The PiB uptake was measured in cortical and cerebellar volumes of interest from frames 50–70 min based on a volume-weighted averaging procedure. To normalize between patients, a cortical/cerebellar uptake ratio was calculated (i.e., global PiB). The cortical SUVR cut-off for defining a subject as amyloid-positive (PiB+) was derived from 93 healthy volunteers and was ≥1.265 using an established method for determination of the cut-off (Vandenberghe et al., 2010). PMOD (PNEURO V3.4) software was used for image analysis.

### Genotyping

Apolipoprotein E genotyping followed standard procedures (Hixson and Vernier, 1990). Participants were classified according to APOE ε4 allele status (carriers: APOEε4+ or non-carriers; APOEε4-).

### Structural and Functional Imaging

MRI data was acquired on a 3 Tesla Ingenia scanner (Philips Healthcare, Best, The Netherlands) using a 15-channel head coil. Headphones and cushions helped to minimize subjects' head motion. Prior to the EEG-fMRI scan, an anatomical T1-weighted 3D-MPRAGE scan was recorded (time of repetition /time of echo: 8.2 ms/3.8 ms, resolution: 1 × 1 × 1 mm, field of view: 240 mm, 170 slices). The fMRI data was recorded with a T2^*^-sensitive multi-slice echo planar imaging sequence (time of repetition = 2.1 s; time of echo = 35 ms; field of view = 230 cm; image matrix = 64 × 63; voxel dimension = 3.6 × 3.6 × 4 mm^3^; slice gap: 0.3 mm, flip angle = 90°, 30 axial slices). One fMRI run comprised 185 volumes (length 2.1 s). Four additional volumes were acquired at the beginning of the sequence, but removed during reconstruction to avoid magnetic saturation effects. Before the fMRI run, a shimming procedure was applied to reduce susceptibility distortions produced by static (local) magnetic field inhomogeneities.

### EEG-fMRI Recording

EEG and fMRI were acquired simultaneously in a 6 min 30sec eyes-closed session (no task) and during various task conditions (here we only report results for the resting state session). Custom made felt cushion were used, filling the space between the electrodes at the back-part of the head. The head was immobilized using foam pads and participants were provided with earplugs. Before each scan, participants were instructed to relax, to refrain from any movements, and to not fall asleep. Participants were personally checked after the anatomical scan (i.e., before the fMRI scan) for tiredness or any indisposition.

### Electroencephalography

EEG recording the scanner were recorded with a 5 kHz sampling rate (32 mV input range, 0.1–250 Hz bandpass filters). Two MR-compatible BrainAmp amplifiers (Brain Products, Gilching, Germany), located outside the scanner room, were used for data transmission via optic fibers. Sixty scalp electrodes were recorded using MR-compatible caps (Easycap, Munich, Germany) with twisted and fixed electrode cables. Electrode positions were from the 10–20 system and additionally from the 10-10 system: FPz, AFz FCz, CPz, POz, Oz, Iz, F5/6, FC1/2/3/4/5/6, FT7/8/9/10, C1/2/5/6, CP1/2/3/4/5/6, TP7/8/9/10, P5/6, PO1/2/9/10, OI1/2). O1/2 and FP1/2 were placed 2 cm next to the standard positions, ensuring an even coverage (Maurer et al., 2007; Brem et al., 2010). F1 was used as recording reference, F2 served as ground electrode. Two electrodes were mounted below the outer canthus of each eye. To record the electrocardiogram, two electrodes were attached right to the sternum and on the left chest close to the heart. Electrode impedances was always <20 kΩ. The EEG signal quality was checked during scanning by an online correction software (RecView, Brain Products, Gilching, Germany).

### Data Post-preprocessing and Analysis Was Perforrocessing: EEG-fMRI

For each subject, EEG preprocessing and analysis was performed with BrainVision Analyzer 2.2 (Brain Products GmbH, Gilching, Germany). Gradient artifacts were eliminated by average artifact subtraction (Allen et al., 1998, 2000), downsampled to 500 Hz and low pass-filtered at 70 Hz cutoff. Ballistocardiogram related artifacts were attenuated using a very similar subtraction method with artifact windows aligned on QRS complexes detected in the electrocardiogram traces (Allen et al., 1998, 2000). First, the EEG was digitally bandpass-filtered (0.5–70 Hz, 24 dB/oct and 50 Hz Notch) and downsampled to 256 Hz. The residual slice artifact at 18 Hz was blocked by a narrow band rejection filter ± 0.5 Hz (Sadaghiani et al., 2010). Using independent component analysis based decomposition (Delorme and Makeig, 2004) with selective back projection, muscle-, movement-, residual gradient-, and ballistocardiogram artifacts were eliminated. Finally, visual inspection was performed to remove any remaining bad intervals and faulty epochs were replaced by neighboring artifact-free EEG epochs (length 2.1 s) to ensure that the total number of epochs was constant for each participant (*n* = 185). Next, channels were transformed to the average reference (Lehmann and Skrandies, 1980).

For each fMRI volume (*n* = 185, length 2.1 s), the respective absolute spectral power was calculated on each channel using a Fast Fourier Transformation (FFT) (Hanning window: 10%, zero padded, 0.5 Hz resolution). Mean absolute spectral band value was computed for delta (1–3 Hz), theta (4–7.5 Hz), alpha1 (8–10 Hz), alpha2 (10.5–13 Hz), and beta (14–30 Hz) bands. The EEG amplitude was calculated as the mean of each FFT-transformed epoch. We computed two EEG outputs for the subsequent EEG-fMRI signal correlations. First, the global spectral power (GSP) (Jann et al., 2009; Michels et al., 2010, 2012; Luchinger et al., 2011, 2012) was calculated for each FFT-transformed epoch as the root mean square across all channel, as a measure of the total oscillatory activity over the scalp. Second, a regional EEG regressors was built in order to account for conventional locally defined EEG rhythms (Luchinger et al., 2011). Here, we focused on the alpha rhythm—as it is the dominant brain rhythm during resting state (Palva and Palva, 2007)—and extracted the mean alpha EEG power from three midline parieto-occipital (Oz, POz, and Pz) electrodes for local alpha EEG-fMRI signal correlation analysis.

### Statistical Analysis: Correlation of Frequency Bands With Bold Signal

All fMRI data were post-processed and analyzed with SPM8 (Wellcome Department of Cognitive Neurology, London, UK). First, images were realigned to the first scan using affine transformation. The realigned images were then normalized into Montreal Neurological Institute (MNI) standard space. Next, all images were resampled to 3 mm cubic voxels, followed by spatial smoothing (8 mm FWHM Gaussian kernel). Voxel-wise EEG-BOLD signal correlations were analyzed by a general linear model (GLM) approach for each band. The model consisted of a boxcar function modeling the eyes closed condition, which was parametrically modulated by the aforementioned GSP-based (or local alpha) EEG power fluctuations. The hemodynamic response function was used to convolve the model regressors. A first-order autoregressive model accounted for serial correlations and a 128 s high-pass filter removed slow signal drifts. For each participant, the GLM contained cerebrospinal fluid, white matter signal as well as the six head motion parameters (nuisance variables). As a result of the GLM, the contrast image of the GSP and regional alpha regressor was written for each subject for subsequent group statistics. For each frequency band (delta, theta, alpha1, alpha2, and beta) and group, we calculated EEG-fMRI signal coupling in positive correlations and anticorrelations.

Group differences were calculated as F-contrasts, i.e., testing for “HCS > < MCI,” to identify all brain regions showing abnormal undirected EEG-fMRI signal coupling. We refrained from running between group *t*-tests, as EEG-fMRI signal correlations are bidirectional, and thus, any stronger correlation in one group could be the result of either a stronger positive in this group or a weaker anticorrelation. However, within-group EEG-fMRI signal correlations were calculated (Supplementary Figure 1), to gain a better insight into the frequency-band specific group differences. Results were reported at height threshold *p* < 0.005, corrected for multiple comparison by extent threshold of minimal cluster size of *k* = 30 voxels (Forman et al., 1995), corresponding to a *p* < 0.05 cluster corrected. The voxel extent threshold based on Monte Carlo simulation using 1,000 permutations (Slotnick et al., 2003). This method was recently validated, revealing acceptable false-positive rates for fMRI inferences (Slotnick, 2017).

While the first analysis set was related to the distinction between HCS and MCI, which is indicative of a disturbed brain function that can be caused by heterogeneous pathologies, we performed a second analysis set focused on the question whether we can identify an EEG- fMRI pattern that is associated with amyloid pathology specifically. Here we compared all PiB+ individuals - MCI-due to AD and HCS—(*n* = 12) to PiB- HCS (*n* = 15). For each analysis, results were only restricted to gray matter volume.

### Bold Signal Spectral Power Analysis

We used the REST toolbox V1.8 (http://restfmri.net) to extract the BOLD signal spectral power from the same preprocessed fMRI images assigned to the EEG-BOLD signal correlation analysis using. Before the analysis, all motion parameters were residualized from the each image. After linear trends removal, ALFF (amplitude of low frequency fluctuation) (Yang et al., 2007; Zang et al., 2007) was calculated for the frequency range of 0.01–0.08 Hz for each participant separately from REST, representing the band average of the square-rooted FFT (taper percent = 0, length = shortest). BOLD spectral power is typically normalized spatially or spectrally to decrease inter-subject variance. Yet, the spatially normalized ALFF [mALFF = ALFF – mean (ALFF)] demonstrates a higher sensitivity to regional differences, and thus, mALFF is the reported measure in our study to illustrate any group difference in BOLD signal amplitude. First, we computed group differences on the whole brain (voxel-to-voxel) level, using two sample unpaired *t*-tests for both contrasts: “HCS > < MCI” and “PiB- > < PiB+” (*p* < 0.005, corrected for multiple comparison by extent threshold with a cluster size of *k* ≥ 30 voxels (Forman et al., 1995), corresponding to a *p* < 0.05 cluster corrected.

As a subsequent analysis, we examined mALFF change in brain regions showing group differences in EEG-fMRI signal coupling for both contrasts. Here, we created a masked image, covering all group differences across all frequency bands (*p* < 0.05, cluster corrected) before running the statistical mALFF group comparison. This analysis was done for two reasons: (1) to minimize multiple comparisons and (2) to link the brain regions showing the strongest BOLD signal and EEG-fMRI signal group difference. All analyses only report fMRI signals differences in gray matter.

## Results

### Sample Characteristics

The sample characteristics of the HCS and MCI is presented in Table 1. Years of education and age was comparable between the 14 MCI and 21 HCS. MCI had lower MMSE scores and scored lower on various cognitive tests. As expected, differences were more pronounced in memory-related scores. Twelve out of 35 subjects were PiB+ (six HCS and six aMCI; Table 1), and 15 HCS were PiB-. In total, nine participants were APOE ε4 carriers (five HCS and four MCI; Table 1).

**Table 1:** Demographic and clinical data for HCS and MCI.

**Group**	**HCS (*n* = 21)**	**MCI (*n* = 14)**	***p*-value**
Global-β-amyloid (Mean/SD)	1.23 (0.2)	1.45 (0.4)	**0.05**
Amyloid-status (PiB+/PiB-)	6/15	6/8	>0.05
ApoE ε4 carrier	5	4	>0.05
Gender (F/M)	7/14	5/9	>0.05
Age in years (Mean/SD)	71.8 (4.2)	75.6 (8.9)	>0.05
Level of education (years)	14.6 (2.9)	14.2 (3.8)	>0.05
**Cognitive variables**			
MMSE, /30	29.6 (0.7)	28.5 (1.6)	**<0.001**
DS (forward)	0.07 (0.8)	−0.4 (0.9)	0.14
DS (backward)	0.61 (1.1)	−0.1 (1.1)	0.07
Trail making (B/A)	−0.12 (1.1)	−0.5 (1.0)	0.71
Letter fluency	0.17 (1.3)	−0.5 (0.8)	0.12
Category fluency	0.51 (1.3)	−0.3 (0.7)	**0.04**
Number of figures	0.3 (1.0)	−0.3 (0.8)	0.09
Stroop Interference	0.52 (0.5)	−0.2 (1.1)	**0.02**
Boston naming	0.32 (0.7)	−0.4 (1.0)	**0.02**
VLMT learning	0.62 (0.6)	−0.9 (0.7)	**<0.001**
VLMT recognition	0.50 (0.7)	−0.7(0.9)	**<0.001**
VLMT delayed recall	0.66 (0.5)	−0.9 (0.7)	**<0.001**
CERAD recall	0.55 (0.6)	−0.8 (0.9)	**<0.001**
CERAD word learning	0.53 (0.8)	−0.7 (0.8)	**<0.001**

*HCS and MCI did not differ in gender distribution (F = female, M = male), age, APOE ε4 (all p > 0.05) but in Aβ deposition (p = 0.05 with MCI > HCS). Cognitive variables are z-transformed raw scores (means and standard deviations) except for MMSE. MCI showed significantly lower test scores for MMSE as well as for category fluency, Stroop interference, Boston naming tests, and – most pronounced – for memory related tests (VLMT and CERAD). MMSE, Mini mental status examination*.

*Bold values indicate significant group differences*.

As shown in Table 2, PiB+ (MCI and HCS combined) demonstrated compared to PiB-(HCS) higher amyloid load, higher presence of APOE ε4+ carriers, lower level of education, as well as lower performance in some neuropsychological test scores, especially in the memory related tests.

**Table 2:** Demographic and clinical data for amyloid positive (PiB+) and amyloid negative (PiB-) individuals.

**Group**	**PiB+ (MCI/HCS)**	**PiB- (HCS)**	***p*-value**
Diagnostic Group	6/6	15	n. a.
Global-β-amyloid (Mean/SD)	1.71 (0.3)	1.1 (0.03)	**<0.001**
ApoE ε4 carrier	4/3	2	n. a.
Gender (F, M)	3/3, 3/3	4/11	n. a.
Age in years (Mean/SD)	74.2 (8.1)	71.1 (3.9)	>0.05
Level of education (years)	12.5 (2.0)	15.3 (3.1)	**0.014**
**Cognitive variables**			
MMSE, /30	28.7 (1.3)	29.6 (0.6)	**0.04**
DS (forward)	−0.39 (0.7)	0.15 (0.8)	0.10
DS (backward)	0.06 (0.8)	0.59 (1.2)	0.22
Trail making (B/A)	−0.52 (1.2)	−0.45 (1.1)	0.90
Letter fluency	0.09 (1.7)	−0.06 (0.9)	0.80
Category fluency	0.52 (1.9)	0.11 (0.8)	0.46
Number of figures	−0.41 (0.8)	0.44 (1.1)	**0.04**
Stroop Interference	−0.35 (1.0)	0.58 (0.5)	**0.007**
Boston naming	−0.25 (1.1)	0.14 (0.7)	0.27
VLMT learning	−0.64 (1.5)	0.67 (0.8)	**0.009**
VLMT recognition	−2.19 (2.0)	0.22 (0.7)	**<0.001**
VLMT delayed recall	−1.51 (1.6)	0.28 (0.8)	**0.001**
CERAD recall	−0.76 (1.5)	0.70 (1.0)	**0.009**
CERAD word learning	−0.13 (1.5)	0.85 (1.2)	0.08

*Groups differ in Aβ deposition (p < 0.001), APOEε4+ presence, gender [female (F)/male (M) distribution], and level of education (p = 0.002). Cognitive variables are z-transformed raw scores means and standard deviations (SD) except for MMSE. PiB+ showed significantly lower performance for MMSE as well as for the Stroop interference task, and for some of the memory related tests (VLMT and CERAD). MMSE, Mini mental status examination*.

*Bold values indicate significant group differences*.

### EEG-fMRI

#### GSP Results

**Figure 1:**
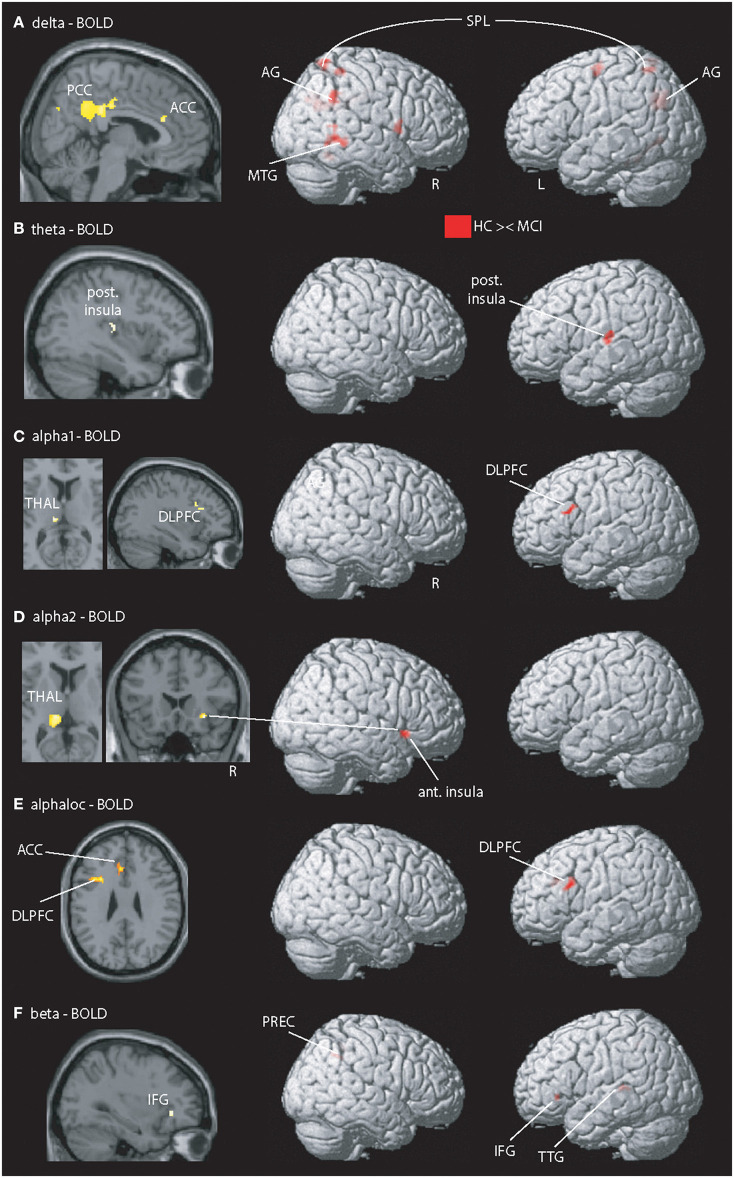
Illustration of MCI-related fMRI signal coupling differences. Rows **(A–D,F)** shows results related to global spectral power (GSP) EEG-fMRI signal coupling; row **(E)** shows the local and band specific EEG-fMRI signal coupling. **(A)** Delta-fMRI signal alterations were seen in the posterior cingulate cortex (PCC), anterior cingulate cortex (ACC), precuneus (PREC), angular gyrus (AG), right frontal operculum, left pallidum, middle temporal gyrus (MTG), left lingual gyrus, fusiform gyrus, bilateral superior parietal lobe (SPL), superior occipital gyrus, precentral gyrus, and bilateral cerebellum exterior. **(B)** In theta, one cluster within the posterior insula (post. Insula) exhibited showed a coupling difference. **(C,D)** Alpha1 and alpha2 revealed altered coupling of the left dorsolateral prefrontal cortex (DLPFC), thalamus (THAL), right fusiform gyrus, and putamen. **(E)** Local alpha-BOLD signal was altered in the left DLPFC and ACC. **(F)** Beta EEG-fMRI signal correlations different in the left frontal operculum covering the inferior frontal gyrus (IFG) and anterior insular cortex (ant. Insula), transverse temporal gyrus (TTG), planum temporale, superior parietal lobe, and precuneus (PREC). For a detailed summary of all coupling results, see Table 2. Results are presented at *p* < 0.05 (cluster-corrected).

Between-group analysis for the contrast “HCS – MCI” (Figures 1A–D,F, Table 3) revealed group-differences in delta EEG-fMRI signal coupling in the PCC (part of the DMN), left precuneus, anterior cingulate cortex (ACC)—frontal component of the DMN –, right central operculum, right middle temporal gyrus (MTG), bilateral superior parietal lobe (posterior part of the FPN), right cuneus and cerebellum exterior. In contrast, theta-BOLD signal coupling was only altered in the left posterior insula [part of the salience network (SAN)]. Alpha1 and alpha2 revealed abnormal alpha1-BOLD signal coupling in the left dorsolateral prefrontal cortex (DLPFC)—a frontal component of the FPN -, left posterior thalamus (alpha1, alpha2), and right anterior insula (alpha 2). Beta EEG-fMRI signal correlations were different in the left inferior frontal gyrus (IFG) and transverse temporal gyrus and right precuneus. Within-group results are shown in Supplementary Figure 1. For example, only HCS showed a (positive) correlation between alpha (and beta) EEG power to the thalamic BOLD signal. In contrast, only MCI demonstrated an anticorrelation between delta to theta EEG power with the fMRI signal of the PCC and frontal regions (IFG, frontal operculum), respectively.

**Table 3:** Summary of differences for between-group EEG-fMRI signal correlations for the contrast “HCS – MCI.”

**Frequency band**	**MNI**	**Area**	***F*-value**
**Delta**			
	2 30 22	Anterior cingulate cortex	17.02
	14−60 70	Superior parietal lobe	14.27
	56−42−6	Middle temporal gyrus	13.73
	−14−60 62	Superior parietal lobe	13.59
	8−32 34	Posterior cingulate cortex	13.13
	48 8 6	Central operculum	11.76
	−6−66 36	Precuneus	11.60
	20−56−16	Cerebellum exterior	10.94
	10−76 34	Cuneus	10.89
**Theta**			
	−34−22 16	Posterior insula	9.69
**Alpha1**			
	−40 16 24	Middle frontal / inferior frontal gyrus	13.02
	2−48 50	Precuneus	12.71
	−34 14 28	Middle frontal gyrus	10.98
	−10−22 10	Thalamus proper	11.17
	56−46−14	Inferior temporal gyrus	10.32
	16−30 8	Thalamus proper	10.16
**Alpha2**			
	−12−26 12	Thalamus proper	17.17
	30 20−8	Anterior insula	13.45
	36−10−4	Posterior insula	13.37
**Beta**			
	24−54 42	Superior parietal lobe	15.39
	−34 32−2	Inferior frontal gyrus / anterior insula	11.17
	−42−30 6	Transverse temporal gyrus	11.01
	2−46 50	Precuneus	10.38

*Results are described using p < 0.05 (cluster-corrected)*.

#### Regional Results

Using selected (Oz, POz, and Pz) electrodes, between-group alpha EEG-fMRI signal coupling differences were present in the (dorsal) ACC and left DLPFC (Figure 1E).

**Figure 2:**
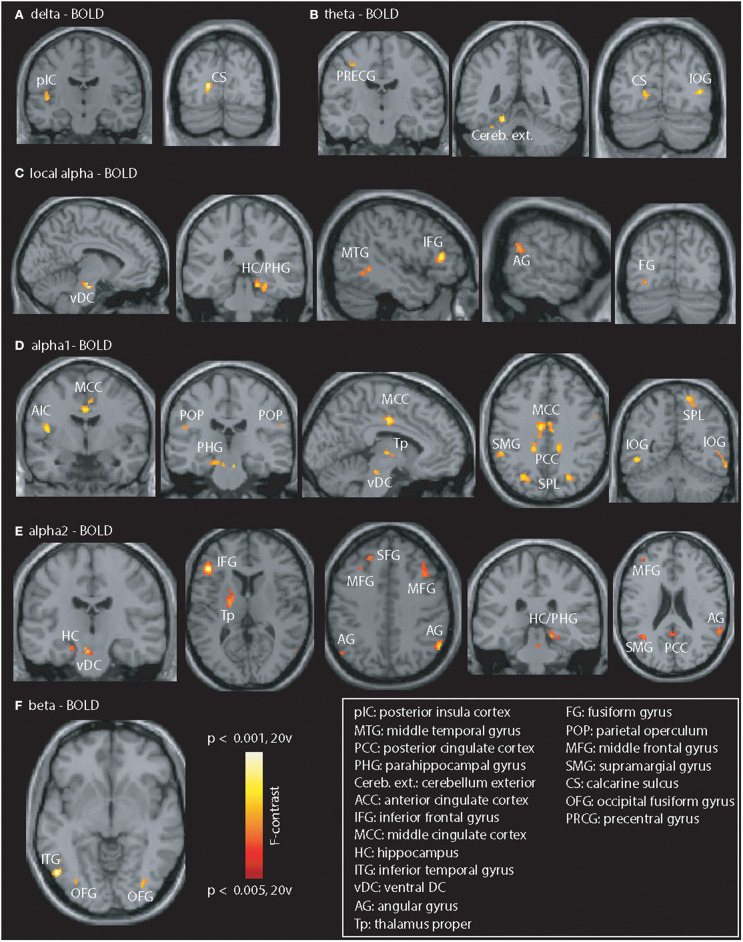
Illustration of PiB-related EEG-fMRI signal coupling differences. Differences were seen in all frequency bands. **(A,B)** Delta / theta: posterior insular cortex (pIC), calcarine sulcus (CS), precentral gyrus (PRCG), cerebellum exterior (Cereb. ext.), and inferior occipital gyrus (IOG). **(C–E)** Local and GSP-based alpha: vDC, ventral DC; HC / PHG, hippocampus / parahippocampal gyrus; MTG, middle temporal gyrus; AG, angular gyrus; IFG, inferior frontal gyrus; FG, fusiform gyrus; AIC, anterior insular cortex; MCC, middle cingulate cortex; POP, parietal operculum; Tp, thalamus proper; PCC, posterior cingulate cortex; SPL, superior parietal lobe; MFG, middle frontal gyrus; SFG, superior frontal gyrus; SMG, supramarginal gyrus. **(F)** Beta: Coupling differences visible in the left ITG, and bilateral fusiform gyrus (OFG). Results are presented at *p* < 0.05 (cluster-corrected).

The contrast “PiB- vs. PiB+” (Figure 2) revealed abnormal coupling in all frequency bands. The lower (delta and theta) bands (Figures 2A,B) mainly showed disturbed coupling in the visual cortex but also in the left posterior insular cortex, precentral gyrus, and cerebellum (exterior). In the local and GSP-derived alpha band (Figures 2C–E), alterations were seen in the visual, frontal, cingulate (not for local alpha power), temporo-parietal regions but also in the thalamus (GSP-derived alpha only) para-hippocampi and brain stem. Beta-fMRI signal coupling (Figure 2F) was abnormal in the inferior temporal and fusiform gyrus.

### Functional MRI

**Figure 3:**
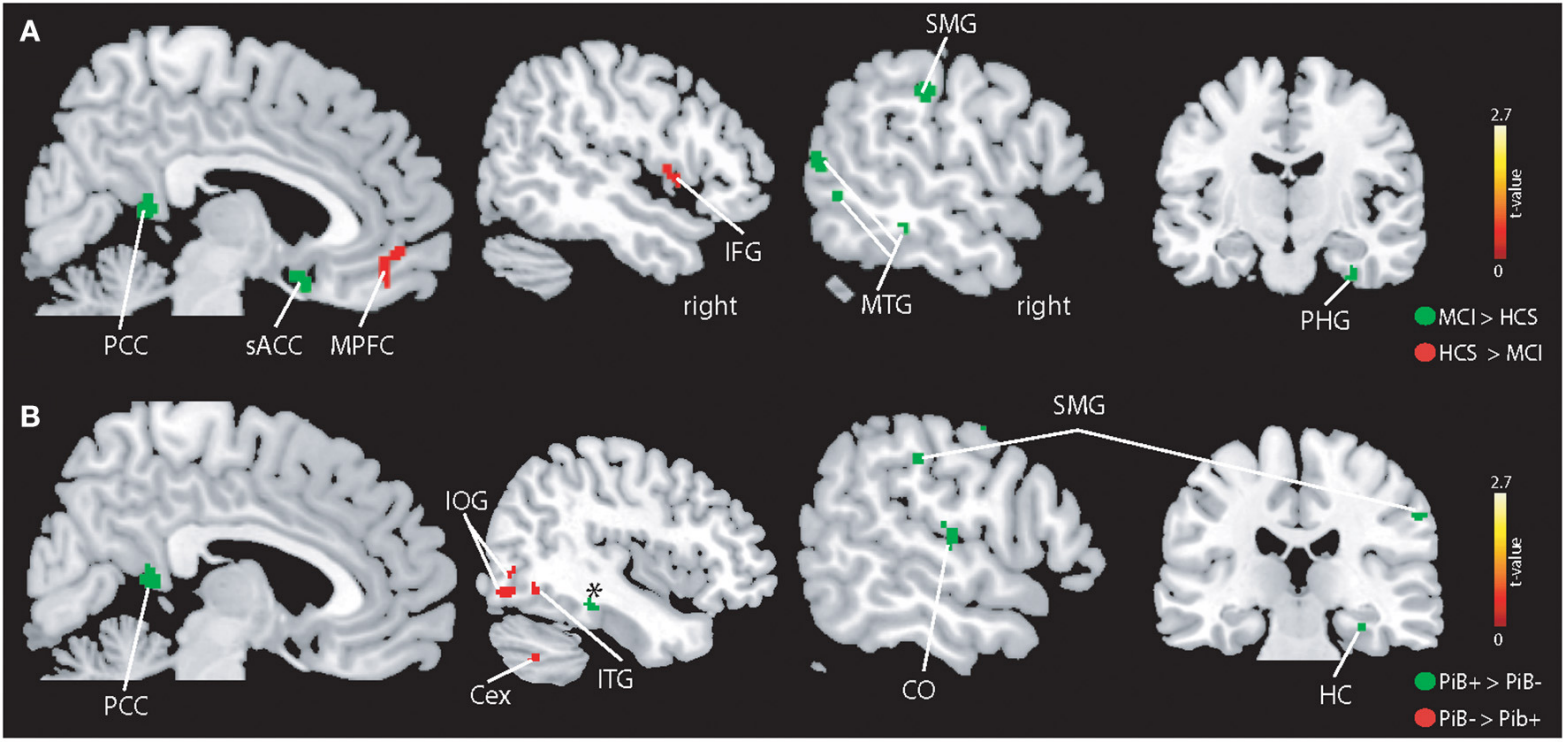
Illustration of the whole-brain mALFF findings. **(A)** Higher BOLD signal amplitudes were seen in patients with MCI compared to controls (indicated in green) in the left supramarginal gyrus (SMG), middle temporal gyrus (MTG), subgenual anterior cingulate cortex (sACC), posterior cingulate cortex (PCC), parahippocampal gyrus (PHG), and other regions (see Results section). HCS showed higher absolute BOLD signal amplitudes in the MPFC and IFG and other regions (see Results section). **(B)** PiB+ individuals demonstrate higher BOLD signals in the hippocampus (HC), central operculum (CO) and PCC. The PiB- group revealed stronger BOLD signal in the inferior temporal gyrus, cerebellum exterior (Cex), and inferior occipital gyrus (IOG). All results are shown at *p* < 0.05 corrected (*t* > 2.7).

On the whole-brain level (Figure 3A), higher BOLD signal was seen in patients with MCI in the left supramarginal gyrus (MNI:−60,−25, 35), posterior cingulate cortex (PCC) (MNI:−3,−45, 11), left MTG (MNI:−57, 62, 9), right parahippocampal gyrus (MNI: 32,−13,−33), left inferior temporal gyrus (MNI:−54,−38,−16), left middle frontal gyrus (MNI:−48, 17, 30), midline sub-genual anterior cingulate cortex (MNI: 4, 11,−16), and right occipital pole (MNI: 21,−98,−7). In contrast, HCS showed stronger BOLD signal in the medial prefrontal cortex (MPFC) (MNI: 8, 42,−12), right frontal operculum (MNI: 47, 4, 5), left middle frontal gyrus (MNI:−28, 47, 30), and left inferior occipital gyrus (MNI:−31,−92, -19).

PiB- demonstrated a stronger BOLD signal (Figure 3B) in the right inferior occipital gyrus (MNI: 39,−80,−9), right cerebellum exterior (MNI: 40,−61,−40). As for patients with MCI, PiB+ revealed higher BOLD signal compared to PiB- in the right parahippocampal gyrus (MNI: 32,−27,−18), right (instead of left) supramarginal gyrus (MNI: 55,−22, 39), PCC (MNI: 4,−48, 15), right (not left) MTG (MNI: 66,−41, 0) and additional brain areas, i.e., right precentral- (MNI: 36,−20, 54) and postcentral gyrus (MNI: 51,−21, 50), and right centro-parietal operculum (MNI: 54,−20, 14).

**Figure 4:**
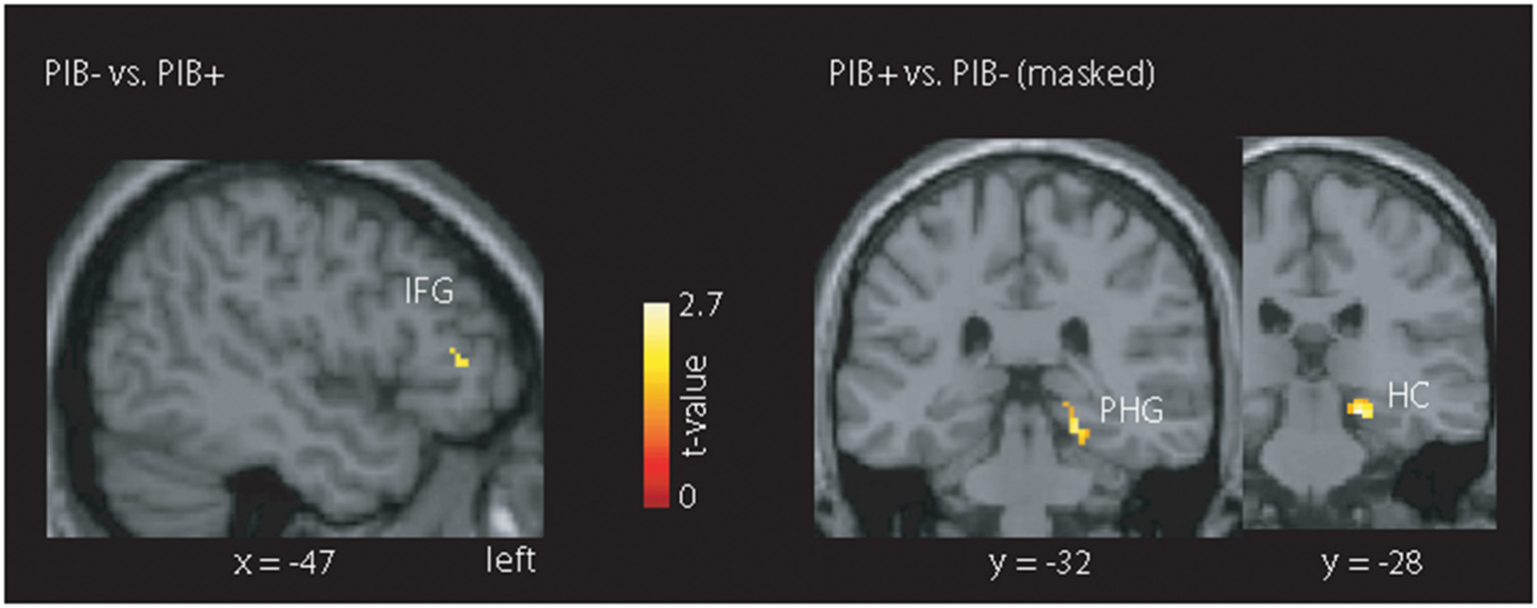
Illustration of the masked mALFF findings. The mask was created across all frequency bands showing an EEG-fMRI signal coupling difference for the contrast “HCS vs. MCI” or the contrast “PiB- vs. PiB+.” Only the latter contrast, we found clusters with stronger absolute BOLD signal for the PiB- group (left inferior frontal gyrus (IFG)) and PiB+ group (right parahippocampal gyrus (PHG) and hippocampus (HC)). All results are shown at *p* < 0.05 corrected (*t* > 2.7).

When we restricted the analysis to regions showing EEG-fMRI signal group differences (across all frequency bands), we did not observe group differences for the contrast “HCS vs. MCI.” For the contrast “PiB- vs. PiB+” (Figure 4), we found heighted fMRI signal responses in the PiB- group in the left inferior frontal gyrus (IFG) (MNI:−50, 34, 10). In contrast, PiB+ showed a higher BOLD signal in a cluster comprising the right parahippocampal gyrus (MNI: 20,−28,−14) and hippocampus (MNI: 14, 36, 2).

## Discussion

The key findings of this study are: (1) EEG-fMRI signal coupling showed a lack of thalamo-cortical coupling in MCI (2) abnormal delta-BOLD signal coupling in the DMN in MCI, (3) abnormal EEG-fMRI signal coupling in PiB+ individuals, especially in hippocampal regions, and (4) altered fMRI signal responses in PiB+ individuals in the lateral prefrontal cortex and hippocampus.

### EEG-fMRI: MCI vs. HCS

Based on developmental EEG-fMRI data, a positive thalamic-BOLD coupling appears to be a marker for brain development (Luchinger et al., 2011, 2012), as it is only consistently present in adults. For example, Laufs et al. and others reported positive thalamic alpha-BOLD signal correlations in healthy adults and inverse correlations in occipital, temporal, and parietal cortex (Goldman et al., 2002; Laufs et al., 2003b; Moosmann et al., 2003; Goncalves et al., 2006; de Munck et al., 2007; Difrancesco et al., 2008; Tyvaert et al., 2008a). Our study is the first demonstrating that a positive thalamic alpha-BOLD signal coupling is also a neurophysiological signature of healthy elderly but not of individuals with MCI, indicative of disturbed thalamo-cortical coupling. Besides alpha, lower frequencies of the EEG power spectrum have also been associated with neural peculiarities of MCI. More specifically, while delta power has been inversely associated with cortical atrophy (Babiloni et al., 2006), both theta band-power and (interhemispheric) coherence could predict the decline from MCI to AD (Rossini et al., 2006). Recently, it has also been shown that instable MCI differ in delta and theta coherence involving the temporal-frontal connections compared to stable MCI (Musaeus et al., 2019). Beason-Held found in a longitudinal study that healthy aging is associated with stable DMN activity measured by [^15^O]PET at two points (Beason-Held et al., 2008). Our findings of abnormal delta and theta EEG-fMRI signal coupling in MCI (Supplementary Figure 1) in parietal (PCC) and frontal (IFG and frontal operculum) brain regions could reflect a neurophysiological impairment in parts of the DMN.

The presence of beta-amyloid-pathology has been associated with altered functional connectivity of the DMN in normal controls (Sperling et al., 2009; Sheline et al., 2010a; Mormino et al., 2011; Quevenco et al., 2020) and in MCI (Sperling et al., 2009). This has also been observed in cognitively healthy elderly individuals without cortical amyloidosis (Sheline et al., 2010b) and in individuals with mild AD but unknown amyloid status (Greicius et al., 2004). As we found abnormal anticorrelations (and BOLD signal) in regions of the DMN, we conclude that patients with MCI display a disturbed neurophysiological coupling in parts of the DMN, In particular, the anticorrelation in the delta band in the MCI group suggests that higher neuronal activity is associated with a decrease in BOLD-fMRI derived signal or vice versa Together with our finding of high BOLD signal in the PCC this could indicate a reduced ability of the neurovascular unit to extract oxygen from the blood. Such an effect might result from a reduced metabolic demand as a consequence of neuronal and synaptic loss together with impaired neurovascular coupling. As in MCI, especially MCI due to AD, hypometabolism has been found in various brain regions including the PCC (Alsop et al., 2000; Chen et al., 2011), we consider this a likely possibility. In such a scenario, despite an overall reduction in CBF as a consequence of less demand, the remaining active neurons would still receive enough oxygen supply. Many pathologies have been described that are associated with a dysfunction of the neurovascular unit including various forms of neurodegenerative diseases as reviewed in Iadecola (2017). However, a recent study found that subjects with MCI display a reduced oxygen extraction fraction compared to elderly controls despite unchanged cerebral blood-flow and cerebrovascular reactivity (Thomas et al., 2017). The authors proposed that in MCI neurons are less able to extract oxygen as a consequence of mitochondrial dysfunction. As we see an anticorrelation of BOLD-fMRI with delta-power such a scenario would be well consistent with our data and could even hint to a pathological mechanism of a mismatch between energy demand and oxygen utilization with the consequence of cognitive impairment. A methodological limitation of our data in this context is that even as we are using EEG-fMRI the exact temporal/causal relationship between neuronal activity and BOLD signal is only feasible in an invasive approach, e.g., in non-human primates (Logothetis et al., 2001).

Another region that showed abnormal (theta) EEG-fMRI signal coupling was the (posterior) insula, which, together with the ACC and supplementary motor area, is part of the SAN. The studies by Sammer et al. (2007) and Mizuhara et al. (2004) commonly reported a positive association between theta-BOLD signal in the hippocampus, superior temporal cortex, cingulate cortex and frontal areas, including the insular cortex, while subjects performed a mental arithmetic task (Mizuhara et al., 2004; Sammer et al., 2007). The posterior insula predominantly connects to the dorsal part of the lateral and central amygdaloid nuclei and connects mutually with the secondary somatosensory cortex. Further, this region has input from the posterior parts of the thalamus. As we examined task-unrelated, i.e. resting-state, EEG-fMRI signal correlations, our results indicate that parts of the SAN (insular cortex) have a disturbed neurophysiological coupling in patients with MCI in a mind-wandering condition that does not require particular attention or cognitive efforts. The SAN is a relevant network, “mediating” between the DMN and the FPN, and thus, disruption of this network could either parallel or even cause the observed abnormalities in the DMN and FPN in this study.

Abnormal alpha EEG/ fMRI signal coupling was seen in the left DLPFC. This region—a frontal part of the FPN—is involved in various executive functions and connect with the orbitofrontal cortex, thalamus, basal ganglia, hippocampus, and primary and secondary association areas (e.g., parietal and occipital areas). Also in the study by Balsters et al., a decrease of coupling was seen in the left FPN (Balsters et al., 2013). Previous fMRI resting-state examinations have produced inconsistent findings related to the extent to which prefrontal cortex activity changes with age. Damoiseaux et al. (2008) could not show age-related differences in the FPN, while Allen et al. (2011) demonstrated significant age-related decreases in BOLD spectra in frontal resting-state networks and the bilateral FPN. Though it has been shown that the FPN showed significant decreases with age (Biswal et al., 2010; Allen et al., 2011), it was the parietal activity and not the prefrontal activity which was reduced. Our findings replicate this observation, as reflected by a significant reduction in alpha1-related coupling of the left FPN, but only in the frontal cortex. Simultaneous EEG/fMRI studies consistently demonstrated correlations between fMRI and beta EEG band power in young adults (Laufs et al., 2003b; Mantini et al., 2007; Balsters et al., 2011) but also in older healthy adults (Balsters et al., 2013) in the DMN. Finally, the observed beta EEG-fMRI signal coupling group differences further underline the abnormal coupling in MCI in parts of the SAN (left insular cortex), FPN (precuneus) but also of the auditory cortex (transverse temporal gyrus).

In a regional (EEG power was taken from midline parieto-occipital electrodes) alpha-band based analysis, Brueggen et al. reported weaker (compared to controls) positive alpha-BOLD signal associations in patients with mild AD in the thalamus, frontal cortex and inferior temporal gyrus in AD patients, and abnormal negative associations in the hippocampus, putamen, and cerebellum (Brueggen et al., 2017). For the HCS-MCI comparison, we could replicate most of the findings, e.g., abnormal coupling in the thalamus (alpha band), frontal cortex (alpha1, local alpha, and beta). However, alterations in the hippocampus and cerebellum were not seen for this comparison. This could be related to the difference in the sample, i.e., MCI vs. patients with mild AD. Yet, focusing on the pathological aspect in our group of subjects, i.e., comparing all PiB+ subjects with PiB- (amyloid negative MCI and HCS), PiB+ additionally demonstrate alterations in the hippocampus and cerebellum, as also seen by Brueggen et al. (2017), and as discussed further below.

### fMRI Signal Alterations

We found abnormally high BOLD signal in patients with MCI in the PCC, ACC, MTG, supramarginal gyrus, and parahippocampal gyrus. A meta-analysis of (12) rs-fMRI studies that reported differences in ALFF (but not mALFF) between aMCI patients and HCS, found that patients with aMCI had—apart from some regional decreases - increased ALFFs in the lingual gyrus, hippocampus, middle occipital gyrus, and inferior temporal gyrus (Pan et al., 2017). This is partially in line with our findings. As for the EEG-fMRI signal findings, our results indicate that some parts of important networks (DMN with PCC, FPN with SMG) demonstrate abnormal high BOLD signal power, whereas other parts of these (DMN with MPFC) and other networks (SAN with IFG) demonstrate abnormally low BOLD signal amplitudes. Strikingly, some of the regions (SMG, PCC, HC) appear not only be altered (in a similar way) by the presence of a cognitive impairment but also by the presence of amyloid when we combine amyloid-positive MCI and HCS thus reflecting the early spectrum of AD. The increase of BOLD signal power in hippocampal regions and adjacent areas (parahippocampal gyrus) could be explained by either a reduced capacity of the brain to extract oxygen from that regions combined with altered neurovascular coupling or hippocampal hypermetabolism. Metabolic alterations in the hippocampus were frequently demonstrated in MCI. A study using task fMRI found that initial hippocampal hyperactivity in MCI and reduction thereof using levetiracetam improved task performance (Bakker et al., 2012). Reduced intrinsic connectivity between hippocampus and precuneus was linked to higher hippocampal glucose metabolism indicating disinhibition like changes of hippocampal activity (Tahmasian et al., 2015). Furthermore, glucose metabolism in the hippocampus was negatively correlated with cognitive performance in a sample of MCI or mild dementia with varying etiological diagnoses thus suggesting rather a detrimental effect than a physiological adaptation (Apostolova et al., 2018). The value of ALFF and related measures (e.g., fractional ALFF) for the identification of individuals with subjective cognitive decline, amnestic MCI, and AD has been shown in a recent machine learning study (Yang et al., 2018). Importantly, the identified regions—leading to group separation—were overlapping with our study, i.e., the ACC, hippocampus, prefrontal brain regions, and more posterior (cingulate) brain regions. The study by Yang et al. reported elevated fMRI signal in the hippocampus in the disease progression groups. Our results add to this finding by demonstrating that also a group of subjects with MCI or PiB+ show this functional alteration. In addition, the hippocampal BOLD signal alteration was still preserved when we focus only on regional clusters, showing an EEG-fMRI signal coupling difference. This indicates that PiB+ participants show an abnormal neurophysiological signature which has its expression in both oscillatory power and fMRI signal alterations.

### The Influence of High Amyloid on EEG-fMRI Signal Coupling

Several brain regions demonstrated amyloid-related whole-brain EEG-fMRI signal coupling and fMRI signal alterations, highlighting the impact of this pathology on oscillatory power and brain function (reflected by the fMRI signal). Most of the coupling differences were seen in deeper brain structures such as the brain stem, cerebellum, hippocampus, parahippocampus, and thalamus, which might not be detectable with (simple) 2D topographic mapping and would require statistical 3D analysis (Michels et al., 2017). Michels et al. (2017) showed that the undirected and directed (effective) EEG alpha and gamma connectivity strength, which was derived from source and not scalp power, was inversely related to amyloid deposition, indicating that amyloid beta load is associated with EEG metrics (Michels et al., 2017). In our study, a new finding was that EEG-fMRI signal coupling partially deviates between the “PiB-contrast” and the contrast focusing on the diagnosis (HCS vs. MCI). For the “PiB-contrast,” we also observe abnormal thalamo-cortical (seen in alpha1/2) and FPN coupling (seen in local and regional alpha bands) but we additionally found consistent (across alpha sub-bands and regional alpha band) coupling alterations in the Pib+ group in the hippocampus and brain stem. The amyloid load is specifically enhanced in the brain stem in late stages of the AD disease, as revealed e.g., by light microscopical analysis (Cole et al., 1993). Our results are not sufficient to directly link local amyloid plaques to neurophysiological alterations. However, the findings could indicate an EEG-fMRI signal effect that reflects an alteration of current along projection axons (originating from the brain stem). Using PiB-PET and FLAIR-MRI, Schreiner et al. (2018) reported a high correlation of (deviated) signal intensity in the hippocampus, basal ganglia and brain stem in cognitively healthy individuals, suggesting that regional amyloid load is associated with tissue edema (Schreiner et al., 2014). Our results indicate that fMRI and EEG-fMRI are sensitive to disease related functional alterations from cortical and subcortical regions in individuals with abnormally-high amyloid levels. The cerebellum is less often reported or discussed (compared to cerebral structures) in fMRI studies of MCI or AD, which is surprising. In fact, an immunohistochemical study demonstrated that cerebellar amyloid-beta plaques—which are primarily of the diffuse type—and amyloid-beta related proteins are less prominent in brains from non-demented patients compared to AD brains (Zhan et al., 1995). Still, some authors reported an equivalent PiB retention (by PiB-PET) by AD and controls in the cerebellum or brain stem, whereas PiB retention was especially high in temporal, frontal, and parietal brain regions in AD (Klunk, 2004; Price et al., 2005), rendering the cerebellum less relevant in AD pathology. Yet, PiB-PET uptake also critically depends on the selection of the scaling region, which is typically the cerebellum, and thus, this region is difficulty to address by PiB-PET. However, it is also known that—even in cognitively healthy individuals - cortical amyloid-beta load had positive effects on cerebro-cerebellar coupling, including regions in the (inferior) temporal gyrus, thalamus, frontal cortex, hippocampus, and parahippocampal gyrus (Steininger et al., 2014). As indicated by our study, we concluded that brain amyloidosis is associated with an abnormal neurophysiological coupling along the cerebro-cerebellar axis.

## Methodological Considerations and Limitations

The simultaneous EEG-fMRI recording does not solve the well-known inverse EEG problem, i.e., multiple (in theory infinite) sets of parameters might explain the same measurement data observed by scalp EEG recordings. In addition, a brain area may have a large metabolic load contributing to the fMRI signal but not to the EEG. For instance, neuronal activity in deep locations can have fMRI correlates that are not measurable by the EEG due to attenuation. Likewise, neuronal activity with tangential orientation to the scalp or opposing and self-canceling sources in sulci will have no correlate in the EEG but can significantly influence the fMRI signal (Nunez and Silberstein, 2000). Stellate cells that are electrically opaque to the EEG as a result of their concentric shape, are mostly GABAergic inhibitory neurons (Connors and Gutnick, 1990). Currently, results are controversial if inhibition is interrelated with negative-, positive-, or no fMRI response (Villringer and Dirnagl, 1995; Logothetis, 2003; Sotero and Trujillo-Barreto, 2007; Sten et al., 2017; Qin et al., 2018; Aksenov et al., 2019). This makes it difficult to directly infer on physiological mechanisms. Still we consider it most likely that the aberrant coupling seen in MCI and PiB+ subjects indicates a mismatch between metabolic demand and supply which could be a consequence of amyloid pathology or an effect resulting in cognitive impairment. Our sample size was small including 14 subjects with MCI, six of which were PiB+ and 21 HCS including six PiB+ subjects. Thus, we refrained from running the analyses for PiB+ HCS and PiB+ MCI separately. However, the sample was well-characterized as all participants underwent a careful neuropsychological testing, multimodal MRI (e.g., Amyloid-PET and EEG-fMRI) and a genetic profiling (APOE4).

## Conclusions

Our results indicate that MCI patients show abnormal cortical and subcortical EEG_power coupling to the BOLD signal. The missing thalamo-cortical and abnormal cortico-cortical coupling indicates a wide-spread metabolic imbalance in individuals with cognitive impairments. Amyloid deposition impairs EEG-fMRI signal coupling especially in hippocampal brain regions and in regions associated with cognitive control and visual processing.

## Data Availability Statement

The datasets presented in this article are not readily available because due to Swiss law, the researchers must assess whether the use of the data and coded datasets are within the primary scope of the informed consent. Subject level data is only available upon request and after the researchers have reviewed the purpose of the inquiry. Requests to access the datasets should be directed to Dr. Lars Michels, lars.michels@usz.ch.

## Ethics Statement

The studies involving human participants were reviewed and approved by the ethics committee of canton Zurich, Switzerland. The patients/participants provided their written informed consent to participate in this study.

## Author Contributions

LM recorded the data, performed the analyses, and drafted the first version of the paper. FR and RM helped with data recording and medical management of the participants. AG was involved in the drafting of the first version of the manuscript. RL and RO'G assisted with the recording of the data. AK and SL performed the neuropsychological assessment. LM, SK, CH, and AG conceptualized and designed the research project. PU, AK, and SL supported the research design. CH conducted the cohort studies as PI together with AG as deputy PI. All authors contributed to the submitted version of the manuscript.

## Conflict of Interest

CH was employed by company Neurimmune AG. The remaining authors declare that the research was conducted in the absence of any commercial or financial relationships that could be construed as a potential conflict of interest.

## Supplementary Material

Supplementary Figure 1 Illustration of within-group EEG-fMRI signal coupling for HCS and MCI. MTG, middle temporal gyrus; SMA, supplementary motor area; THAL, thalamus; PCC, posterior cingulate cortex; MFG, middle frontal gyrus; AG, angular gyrus; STG, superior temporal gyrus; Prec, precuneus; SPL, superior parietal lobe; PCG, precentral gyrus; pINS, posterior insular cortex; INS, anterior insular cortex; FO/IFG, frontal operculum/inferior frontal gyrus; DLPFC, dorsolateral prefrontal cortex; n.s., not significant; R, right hemisphere. Results are presented at *p* < 0.05 (cluster-corrected).

## References

[B1] Aksenov D. P. Li L. Miller M. J. Wyrwicz A. M. (2019). Role of the inhibitory system in shaping the BOLD fMRI response. Neuroimage 201:116034. 10.1016/j.neuroimage.2019.116034 31326573 PMC6886666

[B2] Allen E. A. Erhardt E. B. Damaraju E. Gruner W. Segall J. M. Silva R. F.. (2011). A baseline for the multivariate comparison of resting-state networks. Front. Syst. Neurosci. 5:2. 10.3389/fnsys.2011.00002 21442040 PMC3051178

[B3] Allen P. J. Josephs O. Turner R. (2000). A method for removing imaging artifact from continuous EEG recorded during functional MRI. Neuroimage 12, 230–239. 10.1006/nimg.2000.0599 10913328

[B4] Allen P. J. Polizzi G. Krakow K. Fish D. R. Lemieux L. (1998). Identification of EEG events in the MR scanner: the problem of pulse artifact and a method for its subtraction. Neuroimage 8, 229–239. 10.1006/nimg.1998.0361 9758737

[B5] Alsop D. C. Detre J. A. Grossman M. (2000). Assessment of cerebral blood flow in Alzheimer's disease by spin-labeled magnetic resonance imaging. Ann. Neurol. 47, 93–100. 10.1002/1531-8249(200001)47:1<93::AID-ANA15>3.0.CO;2-8 10632106

[B6] Apostolova I. Lange C. Maurer A. Suppa P. Spies L. Grothe M. J.. (2018). Hypermetabolism in the hippocampal formation of cognitively impaired patients indicates detrimental maladaptation. Neurobiol. Aging 65, 41–50. 10.1016/j.neurobiolaging.2018.01.002 29407465

[B7] Babiloni C. Frisoni G. Steriade M. Bresciani L. Binetti G. Del Percio C.. (2006). Frontal white matter volume and delta EEG sources negatively correlate in awake subjects with mild cognitive impairment and Alzheimer's disease. Clin. Neurophysiol. 117, 1113–1129. 10.1016/j.clinph.2006.01.020 16564740

[B8] Bakker A. Krauss G. L. Albert M. S. Speck C. L. Jones L. R. Stark C. E.. (2012). Reduction of hippocampal hyperactivity improves cognition in amnestic mild cognitive impairment. Neuron 74, 467–474. 10.1016/j.neuron.2012.03.023 22578498 PMC3351697

[B9] Balsters J. H. O'Connell R. G. Galli A. Nolan H. Greco E. Kilcullen S. M.. (2013). Changes in resting connectivity with age: a simultaneous electroencephalogram and functional magnetic resonance imaging investigation. Neurobiol. Aging 34, 2194–2207. 10.1016/j.neurobiolaging.2013.03.004 23608113

[B10] Balsters J. H. O'Connell R. G. Martin M. P. Galli A. Cassidy S. M. Kilcullen S. M.. (2011). Donepezil impairs memory in healthy older subjects: behavioural, EEG and simultaneous EEG/fMRI biomarkers. PLoS ONE 6:e24126. 10.1371/journal.pone.0024126 21931653 PMC3169575

[B11] Beason-Held L. L. Kraut M. A. Resnick S. M. (2008). I. Longitudinal changes in aging brain function. Neurobiol. Aging 29, 483–496. 10.1016/j.neurobiolaging.2006.10.031 17184881 PMC2535938

[B12] Biswal B. B. Mennes M. Zuo X-N. Gohel S. Kelly C. Smith S. M.. (2010). Toward discovery science of human brain function. Proc. Natl. Acad. Sci. U. S. A. 107, 4734–4739. 10.1073/pnas.0911855107 20176931 PMC2842060

[B13] Brem S. Bach S. Kucian K. Guttorm T. K. Martin E. Lyytinen H.. (2010). Brain sensitivity to print emerges when children learn letter-speech sound correspondences. Proc. Natl. Acad. Sci. U. S. A. 107, 7939–7944. 10.1073/pnas.0904402107 20395549 PMC2867899

[B14] Brueggen K. Fiala C. Berger C. Ochmann S. Babiloni C. Teipel S. J. (2017). Early changes in alpha band power and DMN BOLD activity in Alzheimer's disease: a simultaneous resting state EEG-fMRI study. Front. Aging Neurosci. 9:319. 10.3389/fnagi.2017.00319 29056904 PMC5635054

[B15] Chen Y. Wolk D. A. Reddin J. S. Korczykowski M. Martinez P. M. Musiek E. S.. (2011). Voxel-level comparison of arterial spin-labeled perfusion MRI and FDG-PET in Alzheimer disease. Neurology 77, 1977–1985. 10.1212/WNL.0b013e31823a0ef7 22094481 PMC3235355

[B16] Cole G. Neal J. W. Singhrao S. K. Jasani B. Newman G. R. (1993). The distribution of amyloid plaques in the cerebellum and brain stem in Down's syndrome and Alzheimer's disease: a light microscopical analysis. Acta Neuropathol. 85, 542–552. 10.1007/BF00230495 8493862

[B17] Connors B. W. Gutnick M. J. (1990). Intrinsic firing patterns of diverse neocortical neurons. Trends Neurosci. 13, 99–104. 10.1016/0166-2236(90)90185-D 1691879

[B18] Damoiseaux J. S. Beckmann C. F. Arigita E. J. Barkhof F. Scheltens P. Stam C. J.. (2008). Reduced resting-state brain activity in the “default network” in normal aging. Cereb. Cortex. 18, 1856–1864. 10.1093/cercor/bhm207 18063564

[B19] de Munck J. C. Goncalves S. I. Huijboom L. Kuijer J. P. Pouwels P. J. Heethaar R. M.. (2007). The hemodynamic response of the alpha rhythm: an EEG/fMRI study. Neuroimage 35, 1142–1151. 10.1016/j.neuroimage.2007.01.022 17336548

[B20] Debener S. Ullsperger M. Siegel M. Engel A. K. (2006). Single-trial EEG-fMRI reveals the dynamics of cognitive function. Trends Cogn. Sci. 10, 558–563. 10.1016/j.tics.2006.09.010 17074530

[B21] Delorme A. Makeig S. (2004). EEGLAB: an open source toolbox for analysis of single-trial EEG dynamics including independent component analysis. J. Neurosci. Methods 134, 9–21. 10.1016/j.jneumeth.2003.10.009 15102499

[B22] Difrancesco M. W. Holland S. K. Szaflarski J. P. (2008). Simultaneous EEG/functional magnetic resonance imaging at 4 Tesla: correlates of brain activity to spontaneous alpha rhythm during relaxation. J. Clin. Neurophysiol. 25, 255–264. 10.1097/WNP.0b013e3181879d56 18791470 PMC2662486

[B23] Feige B. Scheffler K. Esposito F. Di Salle F. Hennig J. Seifritz E. (2005). Cortical and subcortical correlates of electroencephalographic alpha rhythm modulation. J. Neurophysiol. 93, 2864–2872. 10.1152/jn.00721.2004 15601739

[B24] Folstein M. F. Folstein S. E. McHugh P. R. (1975). “Mini-mental state.” A practical method for grading the cognitive state of patients for the clinician. J. Psychiatr. Res. 12, 189–198. 10.1016/0022-3956(75)90026-6 1202204

[B25] Forman S. D. Cohen J. D. Fitzgerald M. Eddy W. F. Mintun M. A. Noll D. C. (1995). Improved assessment of significant activation in functional magnetic resonance imaging (fMRI): use of a cluster-size threshold. Magn. Reson. Med. 33, 636–647. 10.1002/mrm.1910330508 7596267

[B26] Goldman R. I. Stern J. M. Engel J. Jr. Cohen M. S. (2002). Simultaneous EEG and fMRI of the alpha rhythm. Neuroreport 13, 2487–2492. 10.1097/00001756-200212200-00022 12499854 PMC3351136

[B27] Goncalves S. I. de Munck J. C. Pouwels P. J. Schoonhoven R. Kuijer J. P. Maurits N. M.. (2006). Correlating the alpha rhythm to BOLD using simultaneous EEG/fMRI: inter-subject variability. Neuroimage 30, 203–213. 10.1016/j.neuroimage.2005.09.062 16290018

[B28] Greicius M. D. Srivastava G. Reiss A. L. Menon V. (2004). Default-mode network activity distinguishes Alzheimer's disease from healthy aging: evidence from functional MRI. Proc. Natl. Acad. Sci. U. S. A. 101, 4637–4642. 10.1073/pnas.0308627101 15070770 PMC384799

[B29] Haerting C. Markowitsch H. J. Neufeld H. Calabrese P. Diesinger K. Kessler J. (2000). Wechsler Gedächtnis Test- Revidierte Fassung (WMS-R). Bern: Hans Huber.

[B30] Hampton O. L. Buckley R. F. Manning L. K. Scott M. R. Properzi M. J. Pena-Gomez C.. (2020). Resting-state functional connectivity and amyloid burden influence longitudinal cortical thinning in the default mode network in preclinical Alzheimer's disease. Neuroimage Clin. 28:102407. 10.1016/j.nicl.2020.102407 32942175 PMC7498941

[B31] Hanseeuw B. J. Schultz A. P. Betensky R. A. Sperling R. A. Johnson K. A. (2016). Decreased hippocampal metabolism in high-amyloid mild cognitive impairment. Alzheimers Dement. 12, 1288–1296. 10.1016/j.jalz.2016.06.2357 27421609 PMC5148703

[B32] Hedden T. Van Dijk K. R. Becker J. A. Mehta A. Sperling R. A. Johnson K. A.. (2009). Disruption of functional connectivity in clinically normal older adults harboring amyloid burden. J. Neurosci. 29, 12686–12694. 10.1523/JNEUROSCI.3189-09.2009 19812343 PMC2808119

[B33] Helmstaedter C. H. Lendt M. Lux S. (2001). Verbaler Lern- und Merkfähigkeitstest. Göttingen: Beltz Test.

[B34] Hixson J. E. Vernier D. T. (1990). Restriction isotyping of human apolipoprotein E by gene amplification and cleavage with HhaI. J. Lipid Res. 31, 545–548. 10.1016/S0022-2275(20)43176-1 2341813

[B35] Huijbers W. Mormino E. C. Schultz A. P. Wigman S. Ward A. M. Larvie M.. (2015). Amyloid-beta deposition in mild cognitive impairment is associated with increased hippocampal activity, atrophy and clinical progression. Brain 138, 1023–1035. 10.1093/brain/awv007 25678559 PMC4438387

[B36] Iadecola C. (2017). The neurovascular unit coming of age: a journey through neurovascular coupling in health and disease. Neuron 96, 17–42. 10.1016/j.neuron.2017.07.030 28957666 PMC5657612

[B37] Jacobs H. I. L. Hedden T. Schultz A. P. Sepulcre J. Perea R. D. Amariglio R. E.. (2018). Structural tract alterations predict downstream tau accumulation in amyloid-positive older individuals. Nat. Neurosci. 21, 424–431. 10.1038/s41593-018-0070-z 29403032 PMC5857215

[B38] Jann K. Dierks T. Boesch C. Kottlow M. Strik W. Koenig T. (2009). BOLD correlates of EEG alpha phase-locking and the fMRI default mode network. Neuroimage 45, 903–916. 10.1016/j.neuroimage.2009.01.001 19280706

[B39] Jelic V. Shigeta M. Julin P. Almkvist O. Winblad B. Wahlund L. O. (1996). Quantitative electroencephalography power and coherence in Alzheimer's disease and mild cognitive impairment. Dementia 7, 314–323. 10.1159/000106897 8915037

[B40] Klunk W. E. (2004). Imaging brain amyloid in Alzheimer's disease with Pittsburgh Compound-B. Ann. Neurol. 55, 306–319. 10.1002/ana.20009 14991808

[B41] Laufs H. (2008). Endogenous brain oscillations and related networks detected by surface EEG-combined fMRI. Hum. Brain Mapp. 29, 762–769. 10.1002/hbm.20600 18465797 PMC6871214

[B42] Laufs H. Holt J. L. Elfont R. Krams M. Paul J. S. Krakow K.. (2006). Where the BOLD signal goes when alpha EEG leaves. Neuroimage 31, 1408–1418. 10.1016/j.neuroimage.2006.02.002 16537111

[B43] Laufs H. Kleinschmidt A. Beyerle A. Eger E. Salek-Haddadi A. Preibisch C.. (2003b). EEG-correlated fMRI of human alpha activity. Neuroimage 19, 1463–1476. 10.1016/S1053-8119(03)00286-6 12948703

[B44] Laufs H. Krakow K. Sterzer P. Eger E. Beyerle A. Salek-Haddadi A.. (2003a). Electroencephalographic signatures of attentional and cognitive default modes in spontaneous brain activity fluctuations at rest. Proc. Natl. Acad. Sci. U. S. A. 100, 11053–11058. 10.1073/pnas.1831638100 12958209 PMC196925

[B45] Lehmann D. Skrandies W. (1980). Reference-free identification of components of checkerboard-evoked multichannel potential fields. Electroencephalogr. Clin. Neurophysiol. 48, 609–621. 10.1016/0013-4694(80)90419-8 6155251

[B46] Lim Y. Y. Villemagne V. L. Laws S. M. Ames D. Pietrzak R. H. Ellis K. A.. (2013). BDNF Val66Met, Abeta amyloid, and cognitive decline in preclinical Alzheimer's disease. Neurobiol. Aging 34, 2457–2464. 10.1016/j.neurobiolaging.2013.05.006 23769397

[B47] Logothetis N. K. (2003). The underpinnings of the BOLD functional magnetic resonance imaging signal. J. Neurosci. 23, 3963–3971. 10.1523/JNEUROSCI.23-10-03963.2003 12764080 PMC6741096

[B48] Logothetis N. K. Pauls J. Augath M. Trinath T. Oeltermann A. (2001). Neurophysiological investigation of the basis of the fMRI signal. Nature 412, 150–157. 10.1038/35084005 11449264

[B49] Luchinger R. Michels L. Martin E. Brandeis D. (2011). EEG-BOLD correlations during (post-)adolescent brain maturation. Neuroimage 56, 1493–1505. 10.1016/j.neuroimage.2011.02.050 21349336

[B50] Luchinger R. Michels L. Martin E. Brandeis D. (2012). Brain state regulation during normal development: intrinsic activity fluctuations in simultaneous EEG-fMRI. Neuroimage 60, 1426–1439. 10.1016/j.neuroimage.2012.01.031 22245357

[B51] Mantini D. Perrucci M. G. Del Gratta C. Romani G. L. Corbetta M. (2007). Electrophysiological signatures of resting state networks in the human brain. Proc. Natl. Acad. Sci. U. S. A. 104, 13170–13175. 10.1073/pnas.0700668104 17670949 PMC1941820

[B52] Maurer U. Brem S. Bucher K. Kranz F. Benz R. Steinhausen H. C.. (2007). Impaired tuning of a fast occipito-temporal response for print in dyslexic children learning to read. Brain 130, 3200–3210. 10.1093/brain/awm193 17728359

[B53] Michels L. Bucher K. Luchinger R. Klaver P. Martin E. Jeanmonod D.. (2010). Simultaneous EEG-fMRI during a working memory task: modulations in low and high frequency bands. PLoS ONE 5:e10298. 10.1371/journal.pone.0010298 20421978 PMC2858659

[B54] Michels L. Luchinger R. Koenig T. Martin E. Brandeis D. (2012). Developmental changes of BOLD signal correlations with global human EEG power and synchronization during working memory. PLoS ONE 7:e39447. 10.1371/journal.pone.0039447 22792176 PMC3391196

[B55] Michels L. Muthuraman M. Anwar A. R. Kollias S. Leh S. E. Riese F.. (2017). Changes of functional and directed resting-state connectivity are associated with neuronal oscillations, ApoE genotype and amyloid deposition in mild cognitive impairment. Front. Aging Neurosci. 9:304. 10.3389/fnagi.2017.00304 29081745 PMC5646353

[B56] Michels L. Warnock G. Buck A. Macauda G. Leh S. E. Kaelin A. M.. (2016). Arterial spin labeling imaging reveals widespread and Abeta-independent reductions in cerebral blood flow in elderly apolipoprotein epsilon-4 carriers. J. Cereb. Blood Flow Metab. 36, 581–595. 10.1177/0271678X15605847 26661143 PMC4794091

[B57] Mizuhara H. Wang L. Q. Kobayashi K. Yamaguchi Y. (2004). A long-range cortical network emerging with theta oscillation in a mental task. Neuroreport 15, 1233–1238. 10.1097/01.wnr.0000126755.09715.b3 15167540

[B58] Moosmann M. Ritter P. Krastel I. Brink A. Thees S. Blankenburg F.. (2003). Correlates of alpha rhythm in functional magnetic resonance imaging and near infrared spectroscopy. Neuroimage 20, 145–158. 10.1016/S1053-8119(03)00344-6 14527577

[B59] Mormino E. C. Smiljic A. Hayenga A. O. Ho S. Greicius M. D. Rabinovici G. D.. (2011). Relationships between beta-amyloid and functional connectivity in different components of the default mode network in aging. Cereb. Cortex. 21, 2399–407. 10.1093/cercor/bhr025 21383234 PMC3169663

[B60] Morris J. C. Heyman A. Mohs R. C. Hughes J. P. van Belle G. Fillenbaum G.. (1989). The consortium to establish a registry for Alzheimer's disease (CERAD). Part I. Clinical and neuropsychological assessment of Alzheimer's disease. Neurology 39, 1159–1165. 10.1212/WNL.39.9.1159 2771064

[B61] Musaeus C. S. Nielsen M. S. Hogh P. (2019). Altered low-frequency EEG connectivity in mild cognitive impairment as a sign of clinical progression. J. Alzheimers Dis. 68, 947–960. 10.3233/JAD-181081 30883355

[B62] Nunez P. L. Silberstein R. B. (2000). On the relationship of synaptic activity to macroscopic measurements: does co-registration of EEG with fMRI make sense? Brain Topogr. 13, 79–96. 10.1023/A:1026683200895 11154104

[B63] Palva S. Palva J. M. (2007). New vistas for alpha-frequency band oscillations. Trends Neurosci. 30, 150–158. 10.1016/j.tins.2007.02.001 17307258

[B64] Pan P. Zhu L. Yu T. Shi H. Zhang B. Qin R.. (2017). Aberrant spontaneous low-frequency brain activity in amnestic mild cognitive impairment: a meta-analysis of resting-state fMRI studies. Ageing Res. Rev. 35, 12–21. 10.1016/j.arr.2016.12.001 28017880

[B65] Price J. C. Klunk W. E. Lopresti B. J. Lu X. Hoge J. A. Ziolko S. K.. (2005). Kinetic modeling of amyloid binding in humans using PET imaging and Pittsburgh Compound-B. J. Cereb. Blood Flow Metab. 25, 1528–1547. 10.1038/sj.jcbfm.9600146 15944649

[B66] Qin P. Duncan N. W. Chen D. Y. Chen C. J. Huang L. K. Huang Z.. (2018). Vascular-metabolic and GABAergic inhibitory correlates of neural variability modulation. A combined fMRI and PET study. Neuroscience 379, 142–151. 10.1016/j.neuroscience.2018.02.041 29530810

[B67] Quevenco F. C. Schreiner S. J. Preti M. G. van Bergen J. M. G. Kirchner T. Wyss M.. (2019). GABA and glutamate moderate beta-amyloid related functional connectivity in cognitively unimpaired old-aged adults. Neuroimage Clin. 22:101776. 10.1016/j.nicl.2019.101776 30927605 PMC6439267

[B68] Quevenco F. C. van Bergen J. M. Treyer V. Studer S. T. Kagerer S. M. Meyer R.. (2020). Functional brain network connectivity patterns associated with normal cognition at old-age, local beta-amyloid, tau, and APOE4. Front. Aging Neurosci. 12:46. 10.3389/fnagi.2020.00046 32210782 PMC7075450

[B69] Raichle M. E. MacLeod A. M. Snyder A. Z. Powers W. J. Gusnard D. A. Shulman G. L. (2001). A default mode of brain function. Proc. Natl. Acad. Sci. U. S. A. 98, 676–682. 10.1073/pnas.98.2.676 11209064 PMC14647

[B70] Raichle M. E. Snyder A. Z. (2007). A default mode of brain function: a brief history of an evolving idea. Neuroimage 37, 1083–1090. 10.1016/j.neuroimage.2007.02.041 17719799

[B71] Rane S. Donahue M. J. Claassen D. O. (2018). Amnestic mild cognitive impairment individuals with dissimilar pathologic origins show common regional vulnerability in the default mode network. Alzheimers Dement. 10, 717–725. 10.1016/j.dadm.2018.08.004 30511009 PMC6258224

[B72] Riese F. Gietl A. Zolch N. Henning A. O'Gorman R. Kalin A. M.. (2015). Posterior cingulate gamma-aminobutyric acid and glutamate/glutamine are reduced in amnestic mild cognitive impairment and are unrelated to amyloid deposition and apolipoprotein E. genotype. Neurobiol. Aging 36, 53–59. 10.1016/j.neurobiolaging.2014.07.030 25169676 PMC5531169

[B73] Rossini P. M. Del Percio C. Pasqualetti P. Cassetta E. Binetti G. Dal Forno G.. (2006). Conversion from mild cognitive impairment to Alzheimer's disease is predicted by sources and coherence of brain electroencephalography rhythms. Neuroscience 143, 793–803. 10.1016/j.neuroscience.2006.08.049 17049178

[B74] Sadaghiani S. Scheeringa R. Lehongre K. Morillon B. Giraud A. L. Kleinschmidt A. (2010). Intrinsic connectivity networks, alpha oscillations, and tonic alertness: a simultaneous electroencephalography/functional magnetic resonance imaging study. J. Neurosci. 30, 10243–10250. 10.1523/JNEUROSCI.1004-10.2010 20668207 PMC6633365

[B75] Sammer G. Blecker C. Gebhardt H. Bischoff M. Stark R. Morgen K.. (2007). Relationship between regional hemodynamic activity and simultaneously recorded EEG-theta associated with mental arithmetic-induced workload. Hum. Brain Mapp. 28, 793–803. 10.1002/hbm.20309 17080437 PMC6871320

[B76] Scheeringa R. Bastiaansen M. C. Petersson K. M. Oostenveld R. Norris D. G. Hagoort P. (2008). Frontal theta EEG activity correlates negatively with the default mode network in resting state. Int. J. Psychophysiol. 67, 242–251. 10.1016/j.ijpsycho.2007.05.017 17707538

[B77] Schreiner S. J. Kirchner T. Narkhede A. Wyss M. Van Bergen J. M. G. Steininger S. C.. (2018). Brain amyloid burden and cerebrovascular disease are synergistically associated with neurometabolism in cognitively unimpaired older adults. Neurobiol. Aging. 63, 152–161. 10.1016/j.neurobiolaging.2017.12.004 29310864

[B78] Schreiner S. J. Liu X. Gietl A. F. Wyss M. Steininger S. C. Gruber E.. (2014). Regional Fluid-Attenuated Inversion Recovery (FLAIR) at 7 Tesla correlates with amyloid beta in hippocampus and brainstem of cognitively normal elderly subjects. Front. Aging Neurosci. 6:240. 10.3389/fnagi.2014.00240 25249977 PMC4159032

[B79] Sepulcre J. Sabuncu M. R. Becker A. Sperling R. Johnson K. A. (2013). *In vivo* characterization of the early states of the amyloid-beta network. Brain 136, 2239–2252. 10.1093/brain/awt146 23801740 PMC3692037

[B80] Sheline Y. I. Morris J. C. Snyder A. Z. Price J. L. Yan Z. D'Angelo G.. (2010b). APOE4 allele disrupts resting state fMRI connectivity in the absence of amyloid plaques or decreased CSF Abeta42. J. Neurosci. 30, 17035–17040. 10.1523/JNEUROSCI.3987-10.2010 21159973 PMC3023180

[B81] Sheline Y. I. Raichle M. E. Snyder A. Z. Morris J. C. Head D. Wang S.. (2010a). Amyloid plaques disrupt resting state default mode network connectivity in cognitively normal elderly. Biol. Psychiatry 67, 584–587. 10.1016/j.biopsych.2009.08.024 19833321 PMC2829379

[B82] Slotnick S. D. (2017). Cluster success: fMRI inferences for spatial extent have acceptable false-positive rates. Cogn. Neurosci. 8, 150–155. 10.1080/17588928.2017.1319350 28403749

[B83] Slotnick S. D. Moo L. R. Segal J. B. Hart J. Jr. (2003). Distinct prefrontal cortex activity associated with item memory and source memory for visual shapes. Brain Res. Cogn. Brain Res. 17, 75–82. 10.1016/S0926-6410(03)00082-X 12763194

[B84] Sotero R. C. Trujillo-Barreto N. J. (2007). Modelling the role of excitatory and inhibitory neuronal activity in the generation of the BOLD signal. Neuroimage 35, 149–165. 10.1016/j.neuroimage.2006.10.027 17234435

[B85] Sperling R. A. Laviolette P. S. O'Keefe K. O'Brien J. Rentz D. M. Pihlajamaki M.. (2009). Amyloid deposition is associated with impaired default network function in older persons without dementia. Neuron 63, 178–188. 10.1016/j.neuron.2009.07.003 19640477 PMC2738994

[B86] Steininger S. C. Liu X. Gietl A. Wyss M. Schreiner S. Gruber E.. (2014). Cortical amyloid beta in cognitively normal elderly adults is associated with decreased network efficiency within the cerebro-cerebellar system. Front. Aging Neurosci. 6:52. 10.3389/fnagi.2014.00052 24672483 PMC3957491

[B87] Sten S. Lundengard K. Witt S. T. Cedersund G. Elinder F. Engstrom M. (2017). Neural inhibition can explain negative BOLD responses: a mechanistic modelling and fMRI study. Neuroimage 158, 219–231. 10.1016/j.neuroimage.2017.07.002 28687518

[B88] Tahmasian M. Pasquini L. Scherr M. Meng C. Forster S. Mulej Bratec S.. (2015). The lower hippocampus global connectivity, the higher its local metabolism in Alzheimer disease. Neurology 84, 1956–1963. 10.1212/WNL.0000000000001575 25878180

[B89] Thalmann B. Monsch A. U. Bernasconi F. Berres M. Schneitter M. Ermini-Fuenfschilling D. (1997). CERAD - Consortium to Establish a Registry for Alzheimer's Disease Assessment Battery – deutsche Fassung. Basel: Geriatrische Universitätsklinik.

[B90] Thomas B. P. Sheng M. Tseng B. Y. Tarumi T. Martin-Cook K. Womack K. B.. (2017). Reduced global brain metabolism but maintained vascular function in amnestic mild cognitive impairment. J. Cereb. Blood Flow Metab. 37, 1508–1516. 10.1177/0271678X16658662 27389176 PMC5453471

[B91] Tyvaert L. Hawco C. Kobayashi E. LeVan P. Dubeau F. Gotman J. (2008b). Different structures involved during ictal and interictal epileptic activity in malformations of cortical development: an EEG-fMRI study. Brain 131, 2042–2060. 10.1093/brain/awn145 18669486 PMC3792088

[B92] Tyvaert L. Levan P. Grova C. Dubeau F. Gotman J. (2008a). Effects of fluctuating physiological rhythms during prolonged EEG-fMRI studies. Clin. Neurophysiol. 119, 2762–2774. 10.1016/j.clinph.2008.07.284 18977169 PMC3792084

[B93] Vandenberghe R. Van Laere K. Ivanoiu A. Salmon E. Bastin C. Triau E.. (2010). 18F-flutemetamol amyloid imaging in Alzheimer disease and mild cognitive impairment: a phase 2 trial. Ann. Neurol. 68, 319–329. 10.1002/ana.22068 20687209

[B94] Vannini P. Hedden T. Huijbers W. Ward A. Johnson K. A. Sperling R. A. (2013). The ups and downs of the posteromedial cortex: age- and amyloid-related functional alterations of the encoding/retrieval flip in cognitively normal older adults. Cereb. Cortex 23, 1317–1328. 10.1093/cercor/bhs108 22586140 PMC3643714

[B95] Villringer A. Dirnagl U. (1995). Coupling of brain activity and cerebral blood flow: basis of functional neuroimaging. Cerebrovasc. Brain Metab. Rev. 7, 240–276. 8519605

[B96] Winblad B. Palmer K. Kivipelto M. Jelic V. Fratiglioni L. Wahlund L-O.. (2004). Mild cognitive impairment–beyond controversies, towards a consensus: report of the International Working Group on Mild Cognitive Impairment. J. Intern. Med. 256, 240–246. 10.1111/j.1365-2796.2004.01380.x 15324367

[B97] Yang H. Long X. Y. Yang Y. Yan H. Zhu C. Z. Zhou X. P.. (2007). Amplitude of low frequency fluctuation within visual areas revealed by resting-state functional MRI. Neuroimage 36, 144–152. 10.1016/j.neuroimage.2007.01.054 17434757

[B98] Yang L. Yan Y. Wang Y. Hu X. Lu J. Chan P.. (2018). Gradual disturbances of the amplitude of low-frequency fluctuations (ALFF) and fractional ALFF in Alzheimer spectrum. Front. Neurosci. 12:975. 10.3389/fnins.2018.00975 30618593 PMC6306691

[B99] Zang Y. F. He Y. Zhu C. Z. Cao Q. J. Sui M. Q. Liang M.. (2007). Altered baseline brain activity in children with ADHD revealed by resting-state functional MRI. Brain Dev. 29, 83–91. 10.1016/j.braindev.2006.07.002 16919409

[B100] Zhan S. S. Veerhuis R. Kamphorst W. Eikelenboom P. (1995). Distribution of beta amyloid associated proteins in plaques in Alzheimer's disease and in the non-demented elderly. Neurodegeneration 4, 291–297. 10.1016/1055-8330(95)90018-7 8581561

