# Supplementary figures and images for: EEG-fMRI Signal Coupling Is Modulated in Subjects With Mild Cognitive Impairment and Amyloid Deposition
Source: Front Aging Neurosci. 2021 Apr 23;13:631172. doi: 10.3389/fnagi.2021.631172 (PMC8104007; doi:10.3389/fnagi.2021.631172)

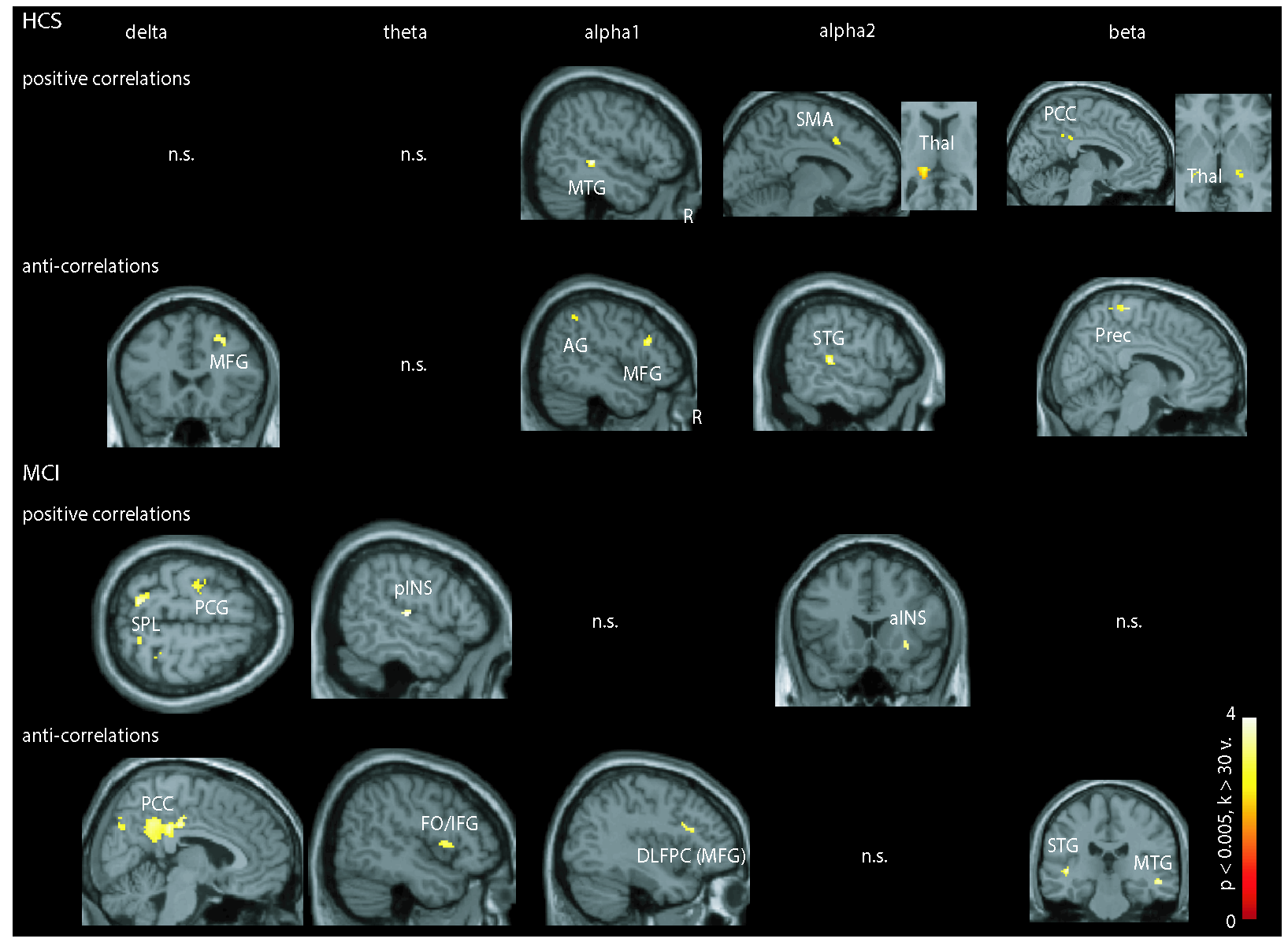

Supplement: Supplementary Figure 1 — Illustration of within-group EEG-fMRI signal coupling for HCS and MCI. MTG, middle temporal gyrus; SMA, supplementary motor area; THAL, thalamus; PCC, posterior cingulate cortex; MFG, middle frontal gyrus; AG, angular gyrus; STG, superior temporal gyrus; Prec, precuneus; SPL, superior parietal lobe; PCG, precentral gyrus; pINS, posterior insular cortex; INS, anterior insular cortex; FO/IFG, frontal operculum/inferior frontal gyrus; DLPFC, dorsolateral prefrontal cortex; n.s., not significant; R, right hemisphere. Results are presented at p < 0.05 (cluster-corrected). [file Image_1.TIF]
